# High Frequencies of *kdr* Mutation and Chikungunya Infection in *Aedes aegypti* Population from Minas Gerais, Brazil

**DOI:** 10.3390/pathogens13060457

**Published:** 2024-05-28

**Authors:** Pedro Augusto Almeida-Souza, Cirilo Henrique de Oliveira, Luiz Paulo Brito, Thaynara de Jesus Teixeira, Iago Alves Celestino, Gabriele Barbosa Penha, Ronaldo Medeiros dos Santos, Wexley Miranda Mendes, Bergmann Morais Ribeiro, Fabrício Souza Campos, Paulo Michel Roehe, Natalia Rocha Guimarães, Felipe C. M. Iani, Ademir Jesus Martins, Filipe Vieira Santos de Abreu

**Affiliations:** 1 Laboratório de Comportamento de Insetos, Instituto Federal do Norte de Minas Gerais, Campus Salinas, Salinas 39560-000, MG, Brazil; pedro.aasouza2020@gmail.com (P.A.A.-S.); cirilohenrique15@gmail.com (C.H.d.O.); thaynarateixeira701@gmail.com (T.d.J.T.); iagoalves958@gmail.com (I.A.C.); gbpenha1@gmail.com (G.B.P.); 2 Programa de Pós-Graduação em Biodiversidade e Uso dos Recursos Naturais, Unimontes, Montes Claros 39401-089, MG, Brazil; 3 Laboratório de Biologia, Controle e Vigilância de Insetos Vetores, Instituto Oswaldo Cruz, Fiocruz, Rio de Janeiro 21040-360, RJ, Brazil; luizpaulobrito2@gmail.com; 4 Departamento de Engenharia Florestal, Instituto Federal do Norte de Minas Gerais, Campus Salinas, Salinas 39560-000, MG, Brazil; ronaldo.medeiros@ifnmg.edu.br; 5 Secretaria Municipal de Saúde de Salinas, Salinas 39560-000, MG, Brazil; wexleymendes@hotmail.com; 6 Laboratório de Baculovirus, Universidade de Brasíla, Brasília 70910-900, DF, Brazil; bergmann@unb.br; 7 Instituto de Ciências Básicas da Saúde, Universidade Federal do Rio Grande do Sul, Porto Alegre 90035-003, RS, Brazil; camposvet@gmail.com (F.S.C.); proehe@gmail.com (P.M.R.); 8 Setor de Arbovirologia, Fundação Ezequiel Dias, Belo Horizonte 30510-010, MG, Brazil; natyroguiman@yahoo.com.br; 9 Instituto Nacional de Ciência e Tecnologia em Entomologia Molecular, UFRJ, Rio de Janeiro 21941-590, RJ, Brazil

**Keywords:** mosquito, arbovirus, pyrethroid, social media, *Culex*

## Abstract

The Chikungunya virus (CHIKV) presents global health challenges, with Brazil experiencing outbreaks since its introduction in 2014. In 2023, following a CHIKV outbreak in Minas Gerais (MG), social media was used to optimize an entomological survey aimed at identifying vectors and viral lineages and assessing insecticide resistance. Following Instagram posts, residents with suspected CHIKV infection were able to schedule mosquito aspirations. In total, 421 mosquitoes (165 *Aedes aegypti* and 256 *Culex quinquefasciatus*) were captured from 40 households in Salinas city (MG) and tested for the Dengue, Zika, and Chikungunya viruses through RT-qPCR. Twelve of 57 pools (10 *Ae. aegypti* and two *Cx. quinquefasciatus*) tested positive for CHIKV RNA. Viral RNA was also detected in the heads of nine *Ae. aegypti*, indicating viral dissemination but not in *Cx. quinquefasciatus*. Genome sequencing yielded the first near-complete genome from the 2023 outbreak, unveiling that the CHIKV strain belonged to the East/Central/South African (ECSA) genotype. Additionally, genetic analyses revealed high frequencies of *kdr* alleles, including in CHIKV-infected mosquitoes, suggesting resistance to pyrethroid insecticides in this *Ae. aegypti* population. Social media was important for guiding mosquito-capture efforts in CHIKV transmission hotspots, thus optimizing the opportunity for viral detection. These findings emphasize the urgent need for innovative vector studies and control strategies, as well as interdisciplinary approaches in public health interventions.

## 1. Introduction

The Chikungunya virus (CHIKV), a member of the *Togaviridae* Family and *Alphavirus* Genus, is an arbovirus responsible for chikungunya fever, a debilitating human disease marked by a high fever and severe joint pain, often leading to long-lasting sequelae [1,2]. First identified during an epidemic in Tanzania, Africa in 1952–1953, CHIKV has since caused outbreaks across Africa, Asia, Europe, and Oceania [3]. Its introduction to the Americas occurred in 2013, reaching Brazil in 2014 and causing explosive outbreaks [4].

In Brazil, the main vector for CHIKV is the *Aedes aegypti* mosquito [5,6]. However, a single genetic mutation (CHIKV E1-226V) has enabled CHIKV adaptation in *Aedes albopictus*, expanding the potential for epidemics in areas with low *Ae. aegypti* infestation [7]. Brazilian populations of *Ae. albopictus* are highly competent at transmitting CHIKV [8,9] and have spread widely since their introduction in 1986 [10,11,12]. Additionally, urban areas in Brazil are infested with *Culex quinquefasciatus*, which has been found to be naturally infected by CHIKV [6,13,14], although its vector competence is controversial [15,16].

The coexistence of these mosquito species in Brazilian cities raises concerns for public health authorities. Despite recommendations for integrated vector-control measures, including chemical control [17], the strategy predominantly relies on insecticides. However, overuse of these chemicals has led to the selection of resistant *Ae. aegypti* populations [18,19]. Although national governmental campaigns have detected resistance to pyrethroids in all Brazilian regions and stopped employing this class of insecticides in 1989 [20], these chemicals are still widely used by households against unwanted indoor insects, including mosquitoes, as they are less irritating to people and produce a rapid knockdown effect. This is likely the main reason for the selection and spread of mutations in the voltage-gated sodium channel gene (NaV), the target site of pyrethroids, known as *kdr* (knockdown-resistant) mutations [21]. There are at least two *kdr* alleles that are widespread in Brazilian *Ae. aegypti* populations, namely NaVR1 and NaVR2, with one (F1534C) and three (V410L, V1016I, and F1534C) mutations [22,23], respectively, with NaVR2 conferring higher levels of resistance [24].

In early 2023, Minas Gerais (MG) experienced its largest Chikungunya (CHIK) outbreak on record, with 69,331 confirmed cases by October, surpassing the totals for 2021 (5,557) and 2022 (13,148) [25]. The northern region of Minas Gerais, characterized by small municipalities with extensive rural areas, was severely affected, reporting 31,410 cases [26]. This region, primarily composed of small municipalities (up to 60,000 inhabitants) [27], faces economic challenges. Despite the magnitude of the outbreak, no entomological studies have been conducted in the region to identify the vectors responsible for transmission. This gap is partly due to a lack of entomologists, logistical challenges, and difficulties in locating mosquitoes, as well as preserving and detecting arboviruses [28].

Hence, this study aimed to conduct an entomological, virological, and genetic investigation to determine the vectors responsible for transmission, infection rates, viral lineage, and presence of insecticide-resistant mutations, taking advantage of social media to optimize the sampling efforts.

## 2. Materials and Methods

### 2.1. Study Area

**Figure 1:**
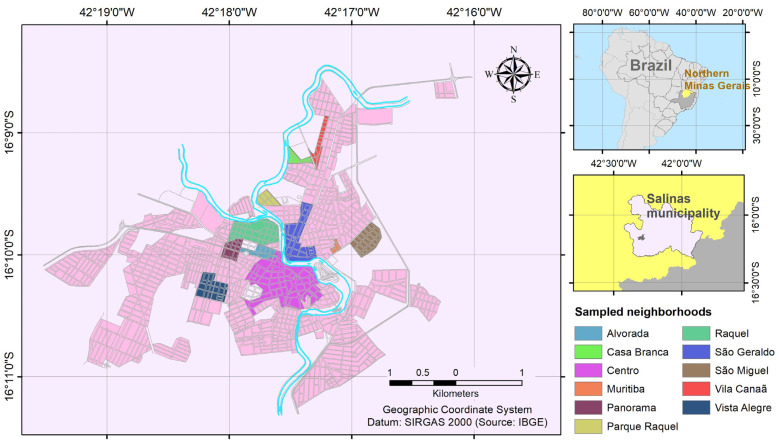
Map showing the northern region of Minas Gerais state, highlighting the city of Salinas and the specific neighborhoods where sampling was conducted.

This study was conducted in the city of Salinas (16°09′45.8″ S; 042°17′54.2″ W), located in the northern region of Minas Gerais (Figure 1). Salinas comprises a population of 40,178 inhabitants and a low municipal human development index (MHDI = 0.679) [29] and is positioned within an ecotone between the Cerrado and Atlantic Forest biomes [30]. The area features a semi-arid climate (Aw climate type according to Köppen, 1936) [31], characterized by two well-defined seasons, namely an extended dry season from March to October and a brief rainy season from November to February, aligning with the periods of sample collection.

### 2.2. Mosquito Collection and Rearing following Instagram Posts

At the onset of 2023, reports of individuals experiencing high fever and arthralgia began circulating in Salinas. By 6 February, eight confirmed cases of CHIK had already been reported in the city [32]. On 8 February, our laboratory’s Instagram profile (@lacoi_ifnmg) issued an announcement regarding our entomological investigation, inviting residents to schedule mosquito aspiration visits (https://www.instagram.com/p/CoarMhNvn_d/?img_index=1; accessed on 20 May 2024). Visits were arranged at the request of residents, based on their availability. Each household was visited by one municipal endemic control agent (a city-hall employee who works on mosquito control) and an entomologist equipped with battery-powered Nasci aspirators [33], oral aspirators, and field entomological cages. Sampling efforts encompassed thorough searches of all rooms within the residences, with particular attention paid to hidden niches, such as under beds and tables and behind sofas and cabinets. Afterward, the number of captured insects was communicated to the residents.

Captured mosquitoes were separated by genera and sex and transferred via oral aspirators to field cages, which were subsequently sealed, labeled, and transported to the Insect Behavior Laboratory at the Federal Institute of Northern Minas Gerais. Within these cages, a 10% sucrose solution soaked in cotton was provided, and the field cages were housed inside larger Bugdorm-type cages (avoiding escape risks) at room temperature (27 ± 4 °C). The mosquitoes were maintained alive for three days to allow blood digestion in potentially engorged females—a period deemed sufficient for the virus to disseminate throughout the mosquito’s body and reach the salivary glands [34]. After three days, the mosquitoes were killed by freezing at −20 °C, transferred to cryovials, and stored in liquid nitrogen (−196 °C) until further processing.

Throughout the study, additional Instagram posts were generated to update the public on the findings and raise awareness about the importance of eliminating mosquito breeding sites (e.g., https://www.instagram.com/p/CpGXmXJvQLg/; https://www.instagram.com/p/CpdonXJv-7u/; and https://www.instagram.com/reel/C22Pl5mu-t-/; accessed on 20 May 2024).

### 2.3. Taxonomic Identification and CHIKV Molecular Diagnosis in the Captured Mosquitoes

**Figure 2:**
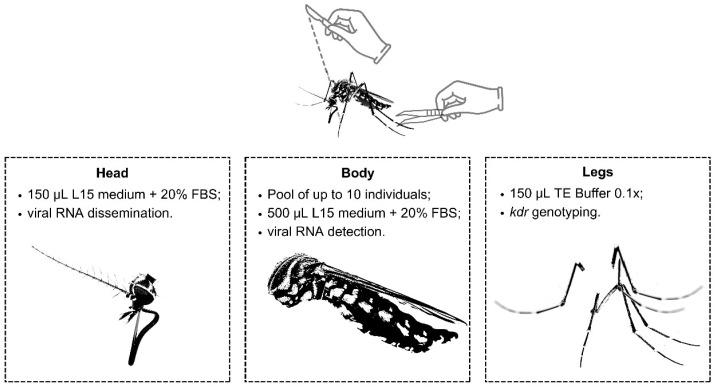
Scheme showing the storage and testing carried out on each anatomical part (head, body, and legs) of the mosquitoes. FBS = fetal bovine serum.

The mosquitoes were transferred from liquid nitrogen and subjected to identification and taxonomic confirmation on a cold table (−20 °C) under a stereoscopic microscope, following dichotomous keys [35,36]. Each mosquito was assigned a unique code and dissected (using individual scalpels to avoid cross-contamination) into three parts: legs, head, and body (Figure 2). The legs were preserved in 150 µL of TE Buffer 0.1×, while the heads were individually stored in tubes containing 150 µL of enriched L-15 medium (20% fetal bovine serum, 0.5% non-essential amino acids, 1% penicillin, 0.1% gentamicin, and 0.1% fungizone) and frozen immediately (Figure 2). Non-engorged mosquito bodies were pooled (up to 10 individuals) by species and sex (Figure 2). These pools were then crushed using a beadbeater (L-Beader 24, Loccus, Cotia, Brazil) in tubes containing beads and 500 µL of enriched L-15 medium (as described above) for 30 s at 7500 rpm [37]. Afterward, these tubes were immediately centrifuged (12,000 rpm, 8 min, 4 °C), and 140 µL of the supernatant was used for RNA extraction using a Qiamp Viral RNA Minikit (Qiagen, Germantown, MA, USA), following the manufacturer’s instructions.

RT-qPCR assays were conducted to detect the presence of Dengue (DENV), Zika (ZIKV), and CHIKV RNA using a ZDC Multiplex PCR Kit (Bioclin Qibasa, Belo Horizonte, Brazil) according to the manufacturer’s instructions. Heads corresponding to insects from positive pools were individually tested to identify the number of infected individuals per pool and assess viral dissemination following the same RNA extraction protocol. Since only CHIKV-positive body pools were obtained, the RNA of the corresponding head samples was subjected to RT-qPCR analysis, as previously described [38]. Briefly, the GoTaq^®^ 1-Step RT-qPCR System (Promega, Madison, WI, USA) was used, along with a specific set of primers and probes targeting the E1 gene, with the following sequences: CHIK F—5′-AAGCTYCGCGTCCTTTACCAAG-3′, CHIK R—5′-CCAAATTGTCCYGGTCTTCCT-3′, and CHIK P—5′-FAM CCAATGTCYTCMGCCTGGACACCTTT-BHQ1-3′ [38]. The RT-qPCR protocol involved reverse transcription at 50 °C for 20 min and initial denaturation at 95 °C for 2 min, followed by 45 cycles of 95 °C for 5 s and 60 °C for 1 min.

### 2.4. CHIKV Genome Sequencing and Phylogenomic Analyses

A representative pool (X-595) was selected, due to its lowest cycle threshold (CT) value, to proceed with whole-genome sequencing. The extracted RNA underwent cDNA synthesis and PCR amplification using a sequencing protocol based on the multiplex PCR-tiling amplicon approach [39]. Subsequently, the resulting amplicons were purified using 1× AMPure XP Beads (Beckman Coulter, Brea, CA, USA) and quantified using a Qubit 3.0 fluorimeter (Thermo Fisher Scientific, Waltham, MA, USA) with a Qubit™ dsDNA HS Assay Kit (Thermo Fisher Scientific). Genomic libraries were then prepared using the Illumina DNA Prep (Illumina, San Diego, CA, USA) and sequenced on the MiSeq platform (Illumina) with v3 (600 cycles) cartridges, following the manufacturer’s instructions.

The sequencing files were processed following an assembly pipeline previously described [40] and publicly available on GitHub (https://github.com/filiperomero2/ViralUnity; accessed on 20 May 2024). Sample genotyping was performed using the Genome Detective Virus Tool, version 2.72 [41]. The newly generated CHIKV genome sequence has been deposited in GISAID under accession number EPI_ISL_19096373.

The new genome was then added to the existing dataset of complete genomes (>11,000 bp) that are publicly available in GenBank for subsequent analysis, resulting in a total of 877 genomes. Sequence alignment was conducted using MAFFT, version 7.490 [42], and visually inspected using AliView, version 1.28 [43]. Maximum-likelihood (ML) trees were generated using IQ-TREE 2.2.5 [44]. The statistical robustness of the tree topology was assessed using 1000 bootstrap replicates.

### 2.5. Analysis of kdr as a Molecular Marker for Pyrethroid Resistance

We utilized the legs of each captured *Ae. aegypti* to individually genotype *kdr* for the three single nucleotide polymorphisms (SNPs) V410L, V1016I, and F1534C. The legs were crushed in a 10% TE solution using two glass beads in Tissue Lyser II (Qiagen) equipment for 2 min at a stirring speed of 30. Subsequently, the samples were homogenized with the addition of 200 µL of TNES and 2 µL of proteinase K (20 mg/mL) and left overnight at 56 °C in a water bath. Following this, 100 µL of 5 M NaCl were added, and the mixture was centrifuged at 15,000× *g* for 6 min. The supernatant was transferred to a new microtube for washing with pure isopropanol, followed by washing with 70% ethanol. Once the pellet was dry, the DNA was resuspended in 50 µL of ultrapure water and stored at −20 °C. We performed independent qPCR reactions for each of the *kdr* SNPs (V410L, V1016I, and F1534C), as described elsewhere [22]. The reactions were carried out in a Thermo Fisher Real-Time Thermocycler, QuantStudio 6 Flex. The obtention of *kdr* genotypic and allelic frequencies considered the variation in the three SNPs of each mosquito (see Souza et al., 2023) [23].

### 2.6. Ethical Statement

The mosquito collection and methods were approved by local authorities (SISBIO-MMA license No. 75826-3; SISGEN No. AF40BCA). This study did not involve endangered or protected species.

## 3. Results

### 3.1. Species Collected, Infection Rates, and Viral Dissemination

Between 8 February and 30 March 2023, we conducted visits to 40 houses across 13 neighborhoods in the city of Salinas, Minas Gerais (Figure 1) that were scheduled through social media. In total, 421 mosquitoes were captured, comprising 256 *Cx. quinquefasciatus* (mean 6.4 ± 6.2 per house) and 165 *Ae. aegypti* (mean 4.1 ± 4.6 per house) (Table 1). Notably, no *Ae. albopictus* specimens were captured during the sampling period (Table 1). The mosquito bodies were grouped into 57 pools, all of which were tested for the presence of DENV, ZIKV, and CHIKV RNA. All pools were negative for DENV and ZIKV. Notably, 12 pools (10 *Ae. aegypti*, including seven female and three male pools; two *Cx. quinquefasciatus*, including one female and one male pool) tested positive for CHIKV (Table 2).

The CT values of the positive pools ranged from 20.1 to 40.0 (Table 2). These 12 CHIKV-positive pools comprised 71 individuals (53 *Ae. aegypti* and *18 Cx. quinquefasciatus*), whose heads were individually examined for the presence of CHIKV to verify viral dissemination. Among these, viral RNA was detected in nine heads, all in *Ae. aegypti* females, from six distinct pools, with CT values below 24.2 (Table 2). Conversely, the heads from the CHIKV-positive pools whose CT values exceeded 28.0, including the *Cx. quinquefasciatus* pools (one male and one female), as well as four *Ae. aegypti* pools (three male and one female), tested negative for CHIKV (Table 2).

### 3.2. CHIKV Genome Sequencing and Phylogenomic Analyses

**Figure 3:**
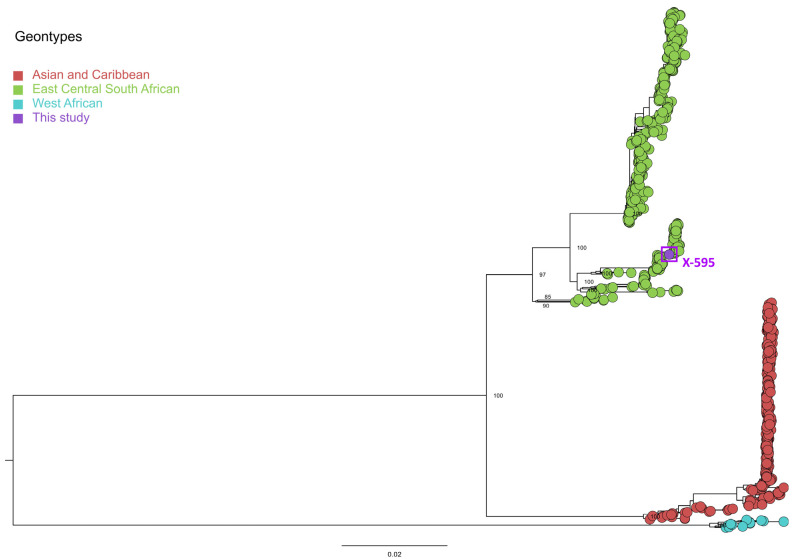
Maximum-likelihood tree of Chikungunya virus genomes inferred using the genome generated in this study and 876 sequences publicly available from GenBank. The scale bar represents the nucleotide substitutions per site (s/s), and the tree is mid-point rooted. The purple circle at the tip represents the genome sequence generated in this study (X-595, GISAIS accession number: EPI_ISL_19096373). Bootstrap values for the major nodes are displayed.

We obtained 247,073 mapped reads for sample x-595, covering 94.8% of the CHIKV genome, with a minimum depth of 20× and an average depth of 2383.88. This represents the first CHIKV genome from MG during the 2023 outbreak, deposited on the gisaid.org platform. The maximum-likelihood (ML) tree grouped these sequences with the CHIKV East/Central/South African (ECSA) genotype, clustering within the same clade as samples detected in humans from São Paulo state, neighboring MG, in 2021 (Figure 3). As expected, the CHIKV E1-226V mutation was not detected.

### 3.3. Analysis of kdr in the Aedes aegypti Population

In total, 164 *Ae. aegypti* mosquitoes were genotyped to verify *kdr* mutations. The most frequent genotype was homozygous for the *kdr* R2 allele, containing the three *kdr* SNPs (LIC), at 40.9%, followed by the heterozygous *kdr* R1/R2 (VVC/LIC) at 32.3% and homozygous for the *kdr* R1 (VVC/VVC) at 11.0% (Table 3). This means that at least 84.2% presented a genotype compatible with pyrethroid resistance, almost half of which would likely display higher levels of resistance (the homozygous *kdr* R2/R2). Out of the 164 mosquitoes genotyped for the three SNPs, only three (1.8%) were homozygous for the wild-type NaVS allele (VVF). Taken together, this reflects an ongoing selection pressure for pyrethroid resistance in *Ae. aegypti* from Salinas. We also observed some uncommonly observed *kdr* genotypes in 2% of the samples, of which the allelic composition and relationship with resistance phenotypes deserve future investigation (Table 3).

Among the nine individuals with detectable CHIKV RNA in their heads, five (55.5%) were R1R2, three (33.3%) R2R2, and one SR2. Although we did not have enough samples to compare the genotypic frequencies between positive and negative CHIKV samples, it was evidenced, for the first time, that there was no constraint for this virus to infect and disseminate in *kdr* mosquitoes.

## 4. Discussion

CHIKV is an arbovirus capable of triggering explosive outbreaks, leading to significant social and economic impacts due to its prolonged clinical manifestations. Due to the absence of a widely available vaccine, vector control remains the primary preventive measure against CHIKV. Therefore, identifying the vectors responsible for virus transmission and assessing insecticide resistance levels are crucial for understanding CHIK epidemiology and implementing effective control measures. In this study, Instagram (a social media platform) proved to be a valuable tool that contributed to the investigation of a CHIK outbreak, highlighting *Ae. aegypti* as the main vector in Minas Gerais, Brazil.

The low diversity of intradomiciliary mosquitoes (*Cx. quinquefasciatus* and *Ae. aegypti* only) is consistent with the essentially urban and anthropophilic habitat of these vectors [5,35]. *Ae. aegypti* is the main vector of urban arboviruses, including Dengue, Zika, and Chikungunya, in Brazil. This species was first documented with natural CHIKV infection in Brazil and the Americas in 2017, attributed to the ECSA genotype [5], which is the same genotype found in the present study. This finding aligns with the widespread prevalence of the ECSA genotype since its introduction in 2014 [45], which is frequently detected in human CHIK cases in Brazil [46,47], including those in the state of Minas Gerais [48]. Despite its importance, there are few reports on genomic CHIKV surveillance in mosquitoes from Brazil [6,12,13,14,49,50,51,52]. This is the first detection of CHIKV in vectors from the southeast of the region, the most urbanized and densely populated area of Brazil.

The elevated MIR observed in *Aedes aegypti* (60.6), coupled with the low CT values obtained, are indicative of high viral RNA loads, underscoring the significant role of this species in the maintenance and transmission of CHIKV within Brazilian urban environments [5,6,49]. Notably, viral RNA was detected in the heads of nine female mosquitoes, indicating viral dissemination and highlighting their potential as vectors. Furthermore, CHIKV RNA was also found in four *Ae. aegypti* male pools, despite the higher CT values, suggesting potential transovarian or sexual transmission mechanisms, which is consistent with findings from previous studies [6,50]. Interestingly, CHIKV RNA was not detected in male heads from positive body pools, nor in female heads from one pool, suggesting limited viral dissemination in these specimens. Previous assessments of CHIKV vector competency have revealed that, despite their high vector competence and viral dissemination in secondary tissues, such as wings and legs, certain individuals’ tissues or saliva may remain uninfected, potentially due to barriers in the midgut or salivary glands, which could impair viral spread [8,9,53].

Despite the greater abundance of *Cx. quinquefasciatus* within households, as previously demonstrated [5,6,49,54], its infection rate (MIR = 7.8) was substantially lower compared to *Ae. aegypti*. Additionally, the CT values were notably high, approaching the assay limit of detection. While Ribeiro Cruz et al. [6] successfully isolated CHIKV from two pools of naturally infected female *Cx. quinquefasciatus* populations, they could not determine vector competence, as the RNA was extracted from whole-body macerates, thus preventing the assessment of possible viral migration to the salivary glands. Consequently, to date, no compelling evidence implicates this species in the transmission of CHIKV in Brazil. Similar to *Ae. aegypti*, the detection of a male *Cx. quinquefasciatus* pool positive for CHIKV RNA suggests the potential for transovarian or sexual transmission, as previously suggested [13,14]. In Kenya, Lutomiah et al. [15] proposed the involvement of *Cx. quinquefasciatus* in CHIKV transmission based on the discovery of naturally infected mosquitoes and the evidence of high vector competency in laboratory assays.

The well-established vector competence of Brazilian *Ae. albopictus* populations for various CHIKV lineages [8,9], along with documented cases of natural infection [14,55] and its widespread distribution across the country [10,56], raises concerns regarding the potential for this species to serve as a vector in Brazil, as observed in other countries [7,57,58]. In Salinas, this species has previously been identified in ovitraps placed in the peridomicile, particularly in residences near the riparian forests of the Salinas River [59]. However, our study did not find *Ae. albopictus* specimens indoors among the 40 sampled residences, suggesting its limited adaptation to indoor environments in this locality. In Brazil, these mosquitoes typically inhabit forest edges in transition areas (ecotones) between forests and urban landscapes, which makes this species a potential bridge vector for arboviruses between these environments [60].

Insecticide resistance is a threat to the control of *Ae. aegypti* globally, making the monitoring of susceptibility a primary necessity for chemical-control sustainability [61]. The surveillance of *kdr* mutations can be used as an indirect indication of pyrethroid resistance, as they partially respond to this phenotype [18]. Based on *kdr* genotyping, herein, we evidenced that the *Ae. aegypti* population from Salinas is probably resistant to pyrethroids, with a high incidence of resistant genotypes. A previous study monitoring *kdr* mutations across Brazil revealed that the *Ae. aegypti* population from Montes Claros (located in the Northern region of Minas Gerais, approximately 170 km away from our study area) already exhibited a high proportion (82.3%) of mosquitoes with resistant genotypes (R1R1, R1R2, and R2R2) [22], quite similar to the 84.2% we found in *Ae. aegypti* from Salinas. Importantly, our research demonstrated that the nine CHIKV-infected individuals presented a *kdr* genotype, indicating that there are no constraints for the dissemination of this virus in *kdr* mosquito organisms. Following the onset of the CHIK outbreak, health authorities implemented an *Ae. aegypti* population-control program, focusing on eliminating breeding sites and utilizing pyrethroid-based insecticides (such as Icon 2.5EW) through ultra-low volume spraying. Additionally, during mosquito sampling, we observed the widespread use of pyrethroid-based commercial insecticides in many of the visited households. The sustained use of pyrethroids has likely exerted selective pressure on resistant alleles/genotypes, thereby reducing the effect of chemical-control efforts and potentially leading to the enhancement of outbreaks. Collectively, our findings underscore the imperative to invest in novel vector-control strategies (such as *Wolbachia*-infected and transgenic mosquitoes), in increased efforts to stimulate entomological surveillance (including digital tools and citizen science initiatives), and in vaccine research to mitigate the risk of arboviral disease outbreaks. In this regard, it is noteworthy that a CHIKV vaccine was recently approved in the United States [62], which could be a crucial tool for prevention.

The use of smartphone applications and social media platforms has recently emerged as a pivotal tool for mosquito surveillance, vector-borne disease monitoring, and scientific knowledge dissemination [63,64,65,66,67,68,69,70,71]. In this study, the utilization of a social media platform improved collection efforts by guiding collections at potential CHIKV transmission hotspots, thereby optimizing resources and increasing the probability of detecting arboviruses. The use of social media plays a vital role in scientific dissemination, facilitating closer engagement between the research community and the general public. Given the recognized limitations of conventional arbovirus surveillance and control methods, the adoption of these innovative tools and technologies is becoming increasingly imperative.

While our study provided valuable new findings, it is crucial to acknowledge its limitations. First, the focus on entomological investigations within households may not have fully captured the diversity and dynamics of vector populations in peri-domestic and sylvatic environments. Additionally, while the use of a social media platform facilitated the identification and aspiration of houses with suspected arboviral cases, relying solely on such platforms may introduce selection biases, as individuals with access to and are familiar with these platforms may differ from those who do not participate. Lastly, the geographical scope of this study was confined to one municipality in Minas Gerais, Brazil, limiting broader generalizations about CHIKV vector ecology and insecticide resistance patterns across different regions. Despite these limitations, our findings underscore the importance of innovative approaches, such as social media-driven citizen science, in bolstering entomological surveillance efforts and advancing our comprehension of arbovirus epidemiology and control strategies.

## 5. Conclusions

In conclusion, our study underscores the crucial role of entomological surveillance in comprehending the epidemiology and control of CHIKV infections. Through employing innovative technologies, including social media, we identified *Ae. aegypti* as the primary vector of CHIKV (ECSA genotype) during the 2023 outbreak in Minas Gerais, Brazil. The high frequency of *kdr* mutations—indicative of pyrethroid resistance in the vector population—was also revealed, even among CHIKV-infected individuals, suggesting that there is no barrier for this virus to infect and disseminate within *kdr* mosquitoes. Despite inherent limitations, such as the focus on domestic areas and potential selection bias, our findings emphasize the urgent necessity for innovative vector-control strategies and the development of novel vaccines to effectively mitigate arboviral disease outbreaks. Additionally, we highlight the growing importance of interdisciplinary approaches, including collaboration among researchers, public health professionals, and community members, in order to bolster entomological surveillance and address the ongoing challenges posed by mosquito-borne diseases.

## Figures and Tables

**Table 1:** Captured mosquitoes and presence of CHIKV RNA in Salinas, MG, Brazil.

Species	Male	Female	Sum (Relative Abundance %) ^#^	Pools Tested (CHIKV-Positive)	MIR *
*Ae. aegypti* (Linnaeus, 1762)	70	95	165 (39.2)	31 (10)	60.6
*Cx. quinquefasciatus* Say, 1823	143	113	256 (60.8)	26 (2)	7.8
Total	213	208	421 (100)	57 (12)	28.5

* Minimum infection rate (MIR) = No. of positive pools/No. of same species adults analyzed × 1000; ^#^ relative abundance = No. of insects of each species/total No. of insects.

**Table 2:** Description of CHIKV-positive pools and corresponding individual heads tested.

Cod. Pool	Species	No. of Individuals	Sex	CT *	Individual Heads	Positive Heads (CT **)
X-595	*Ae. aegypti*	5	F	20.1	c144, c145, c146, c147, c148	c144 (21.5); c146 (22.0); c148 (30.1)
X-556	*Ae. aegypti*	5	F	21.0	c33, c34, c35, c36, c37	c34 (21.2)
X-585	*Ae. aegypti*	5	F	22.9	c82, c83, c84, c88, 89	c83 (23.6); c84 (35.8)
X-584	*Ae. aegypti*	2	F	23.1	c81, c108	c81 (21.6)
X-579	*Ae. aegypti*	5	F	24.1	c51, c52, c54, c56, c58	c58 (26.9)
X-594	*Ae. aegypti*	5	F	24.2	c139, c140, c141, c142, c143	c141 (23.0)
X-606	*Ae. aegypti*	5	F	28.0	c149, c150, c151, c152, c164	_
X-555	*Ae. aegypti*	6	M	38.0	c30, c32, c39, c40, c43, c44	_
X-593	*Ae. aegypti*	7	M	38.3	c126, c128, c134, c135, c136, c137, c138	_
X-586	*Ae. aegypti*	8	M	40.0	c85, c86, c87, c90, c91, c92, c93, c94	_
X-623	*Cx. quinquefasciatus*	9	M	38.1	c296, c297, c298, c299, c300, c301, c302, c303, c304	_
X-619	*Cx. quinquefasciatus*	9	F	38.2	c254, c255, c256, c257, c258, c259, c260, c261, c262	_

* CT of reactions with the abdomen pools; ** CT of reactions with each individualized head from their respective positive pools. The symbol “–” means that no CHIKV RNA was detected in the heads, suggesting a lack of viral dissemination.

**Table 3:** NaV genotypes in *Ae. aegypti* from Salinas, MG, Brazil.

Genotypes	VV+VV+ FF	VV+VV+ FC	VV+VV+ CC	VL+VI+ FC	VL+VI+ CC	LL+II+ CC	VV+Vl+ CC *	VV+VI+ FC	VL+II+ CC
No. of individuals (Frequency)	3	2	18	17	53	67	2	1	1
	(1.8)	(1.2)	(11.0)	(10.4)	(32.3)	(40.9)	(1.2)	(0.6)	(0.6)

Composite genotype for the three SNPs: 410 + 1016 + 1534, composed of the allele NaVS (VVF) and the *kdr* R1 (VVC) and R2 (LIC). * Uncommon genotypes.

## Data Availability

All data are shown in the manuscript.
